# Ruxolitinib, a JAK1/2 Inhibitor, Ameliorates Cytokine Storm in Experimental Models of Hyperinflammation Syndrome

**DOI:** 10.3389/fphar.2021.650295

**Published:** 2021-04-22

**Authors:** Eduardo Huarte, Michael T. Peel, Katherine Verbist, Brittany L. Fay, Rachel Bassett, Sabrin Albeituni, Kim E. Nichols, Paul A. Smith

**Affiliations:** ^1^ Incyte Research Institute, Wilmington, DE, United States; ^2^ St. Jude Children’s Research Hospital, Memphis, TN, United States

**Keywords:** hyperinflammatory syndromes, cytokine release syndrome, cytokine storm syndrome, hemophagocytic lymphohistiocytosis, JAK inhibition

## Abstract

Hyperinflammatory syndromes comprise a heterogeneous group of disorders characterized by severe inflammation, multiple organ dysfunction, and potentially death. In response to antigenic stimulus (e.g., SARS-CoV-2 infection), overactivated CD8+ T-cells and macrophages produce high levels of proinflammatory cytokines, such as IFN-γ, TNF-α, IL-6, and IL-12. Multiple inflammatory mediators implicated in hyperinflammatory syndromes utilize the Janus kinase–signal transducers and activators of transcription (JAK-STAT) cascade to propagate their biological function. Our findings demonstrate that oral ruxolitinib dosing designed to mimic clinically relevant JAK-STAT pathway inhibition significantly reduces the harmful consequences of immune overactivation in multiple hyperinflammatory models. In contrast to monoclonal antibody therapies targeting a single cytokine, ruxolitinib effectively downregulates the functional effect of multiple cytokines implicated in hyperinflammatory states, without broad immunosuppression.

## Introduction

The worldwide surge in morbidity and mortality due to hyperinflammatory syndromes brought by the COVID-19 pandemic has rekindled an interest in such pathophysiologies (Cao et al., 2020; Kale et al., 2020; Mangalmurti and Hunter, 2020). Hyperinflammatory syndromes, mainly cytokine storm syndrome (CSS) and cytokine release syndrome (CRS), are severe conditions characterized by the excessive activation of immune cells (T-cells and macrophages) and cytokine production leading to multiorgan failure and often death. While CSS and CRS share many clinical characteristics, there are important differences in the pathophysiology. CSS is observed after viral infections (Tisoncik et al., 2012), and acute respiratory distress syndrome (ARDS) constitutes the leading cause of mortality (Mehta et al., 2020b). CSS may also be associated with macrophage activation syndrome and hemophagocytic lymphohistiocytosis (HLH), with overlapping physiologies (Behrens and Koretzky, 2017). Patients with CSS are frequently refractory to the current standard of care (glucocorticoids and the chemotherapeutic agent etoposide), resulting in a clear clinical need to develop new therapeutic agents to limit inflammation, reduce cytokine levels, and ultimately prevent multiorgan failure and death (Meyer et al., 2020). In contrast, CRS is the result of an overactivation of immune cells often seen in the aftermath of immunotherapies such as chimeric antigen receptor T-cells (CAR-T) or bispecific antibodies (Lee et al., 2014; Huarte et al., 2020).

JAKs play an important role in signal transduction following cytokine and growth factor binding to their cognate cell surface receptors. Upon activation, JAKs phosphorylate transcription factor STATs, which results in dimerization and translocation of STATs to the nucleus to activate gene transcription and initiate an inflammatory response (Howell et al., 2019). Multiple cytokines implicated in hyperinflammatory syndromes (e.g., IL-2, IL-6, IL-7, IL-10, IFN-γ and G-CSF) rely on the JAK/STAT pathway. We and others have suggested the use of JAK inhibitors in several hyperinflammatory conditions (Meyer et al., 2020). ruxolitinib, an equipotent and selective JAK1 and JAK2 inhibitor (Quintás-Cardama et al., 2010), is FDA approved for the treatment of myelofibrosis, polycythemia vera, and acute steroid-refractory graft-versus-host-disease. Ongoing clinical trials are exploring the therapeutic potential of ruxolitinib in HLH (NCT04414098, NCT04355793 and NCT04551131) (Ahmed et al., 2019).

Here, we elucidate the effect of ruxolitinib at clinically achievable JAK/STAT pathway inhibition using several *in vitro* and *in vivo* models of hyperinflammation, including CRS and HLH, both primary [i.e., genetic] as well as secondary [i.e., non-genetic]. Our data show that ruxolitinib, but not anti-IL-1R nor anti-IL-6R monoclonal antibodies (mAb), significantly reduces cytokine expression by both T-cells and macrophages and ameliorates disease symptoms, thus supporting the hypothesis that generalized JAK/STAT pathway activation plays a critical role in hyperinflammatory syndromes and that its pharmacological inhibition may represent a viable therapeutic strategy.

Overall, ruxolitinib may represent a therapeutic intervention to address the need for more effective treatments for patients developing hyperinflammatory syndromes.

## Methods

### Animals

C57BL/6 and BALB/c mice were purchased from Taconic Biosciences (Rensselaer, NY, United States) and were approximately 8 weeks old. Ovalbumin (OVA) transgenic TCR mice (OT-1) and perforin deficient mice (*Prf1* ^−/−^) were purchased from the Jackson Laboratory (Bar Harbor, ME, United States). All mice were used in protocols approved by the Institutional Animal Care and Use Committees at the respective institutions.

### Ruxolitinib Modulation of Mouse Cytokine Release Syndrome Models

CRS was induced in BALB/c mice by ConA injection (20 mg/kg, IV). Animals were dosed with ruxolitinib (30 or 60 mg/kg, PO), anti-IL-1R mAb (25 mg/kg, IP) or anti-IL-6R mAb (25 mg/kg, IP) 60 min before (prophylactic) or 30 min after (therapeutic) CRS induction. Two hours after ConA injection, mice were sacrificed and serum was collected for cytokine measurement.

### Hemophagocytic Lymphohistiocytosis Mouse Models

For the secondary HLH model, C57BL/6 mice were injected IP with 50 μg of CpG DNA on days 0, 2, 4, 6, and 8. Starting on day 5, mice were treated with vehicle (PO, BID), ruxolitinib (60 mg/kg, PO, BID), anti-IL-1R mAb (25 mg/kg, IP, QD), or anti-IL-6R mAb (25 mg/kg, IP, QD). In the primary (genetic) model of HLH, *Prf1* ^*−/−*^ mice were IP infected with 2 × 10^5^ PFU LCMV Armstrong and treated with control or ruxolitinib chow (2 g/kg) starting on day 4 post-infection (Meyer et al., 2020).

### Transcriptomic Analysis

Splenic T-cells were isolated using a pan T-cell isolation kit and an autoMACS Pro Separator (Miltenyi Biotec, Bergisch Gladback, Germany). Approximately 2 × 10^6^ cells were lysed in 500 µl Trizol (Invitrogen, Carlsbad, CA, United States) in gentleMACS M tubes. RNA was purified using a Trizol Plus RNA purification kit (Invitrogen). 100 ng of RNA was hybridized with the nCounter mouse immunology panel codeset (NanoString Technologies, Inc. Seattle, WA, United States) for 18 h. The cartridges were run on an nCounter SPRINT profiler (NanoString Technologies, Inc.). Data were analyzed using nSolver 4.0 Advanced Analysis software. *p* values were adjusted using the Benjamini-Hochberg method.

### T-Cell Proliferation Assay

Splenocytes were incubated with CFSE to permit measurement of proliferation. T-cells were activated with Dynabeads (Thermo Fisher Scientific) at a 3:1 ratio, resuspended at a density of 0.5 × 10^6^ cells/mL in 24-well plates, and treated with ruxolitinib at various concentrations. The plates were incubated for 7 days, and proliferation was determined by flow cytometry.

### CD107a Degranulation Assay

Splenocytes from OT-1 or C57BL/6 were resuspended at 5 × 10^6^ cells/mL in complete RPMI, 20 IU IL-2, anti-CD3 (5 μg/ml, plate bound) and anti-CD28 (1 μg/ml) antibodies, and increasing ruxolitinib concentrations. After 3–5 days, OT-1 cells were collected and stained overnight with an anti-CD107a antibody. The cultures were then incubated with OVA^+^ EG-7 tumor cells for 5 h at 37°C. Immediately following stimulation, cultures were washed once, surface stained with directly conjugated antibodies against CD3 and CD8, and analyzed by flow cytometry.

### Activated Macrophage Models

Bone marrow was collected from C57BL/6 mice, and bone marrow cells were cultured in RPMI medium supplemented with 10% fetal bovine serum and 10 ng/mL M-CSF. On day 6, cells were treated with varying ruxolitinib concentrations and then incubated with 2.5 ng/ml lipopolysaccharide (LPS) on day 7. On day 8, cytokines were measured from supernatants. For the *in vivo* model, C57BL/6 mice were prophylactically dosed with vehicle, ruxolitinib (60 mg/kg, PO), anti-IL-1R mAb (25 mg/kg, IP), or anti-IL-6R mAb (25 mg/kg, IP). Mice were then challenged with LPS (5 µg per animal). Two hours after LPS injection, mice were euthanized, and a peritoneal lavage was performed.

### Cytotoxicity Assay

Splenocytes from OT-1 mice were incubated in the presence of 2 µg/ml of the ovalbumin peptide, SIINFEKL, for 3 days. During this time, OVA-expressing EG-7 cells were transfected with a pGL3 luciferase plasmid (Promega, E1751) using lipofectamine 2000 (ThermoFisher, 11,668,030) according to manufacturer’s instructions. After 3 days, OT-1 cells were mixed with EG-7 target cells in a 5:1 ratio and incubated at 37° for 5 h. Following incubation, 50 µL of Bright-Glo luciferase reagent (Promega, E2610) was added to the cultures and fluorescence was measured by plate reader.

### Data and Statistical Analysis

Data are reported as mean + SEM in the relevant figures. Differences between groups were analyzed by nonparametric Mann-Whitney test. Statistical analysis for multiple groups was performed by Kruskal-Wallis with Dunn’s post hoc test for nonparametric data sets or analysis of variance with Holm-Sidak’s test for parametric results. All tests were performed using GraphPad Prism (GraphPad Software Inc., San Diego, CA, United States).

## Results and Discussion

### Ruxolitinib Reduces Exaggerated Cytokine Levels in Murine Models of Acute Hyper-Inflammation

**FIGURE 1:**
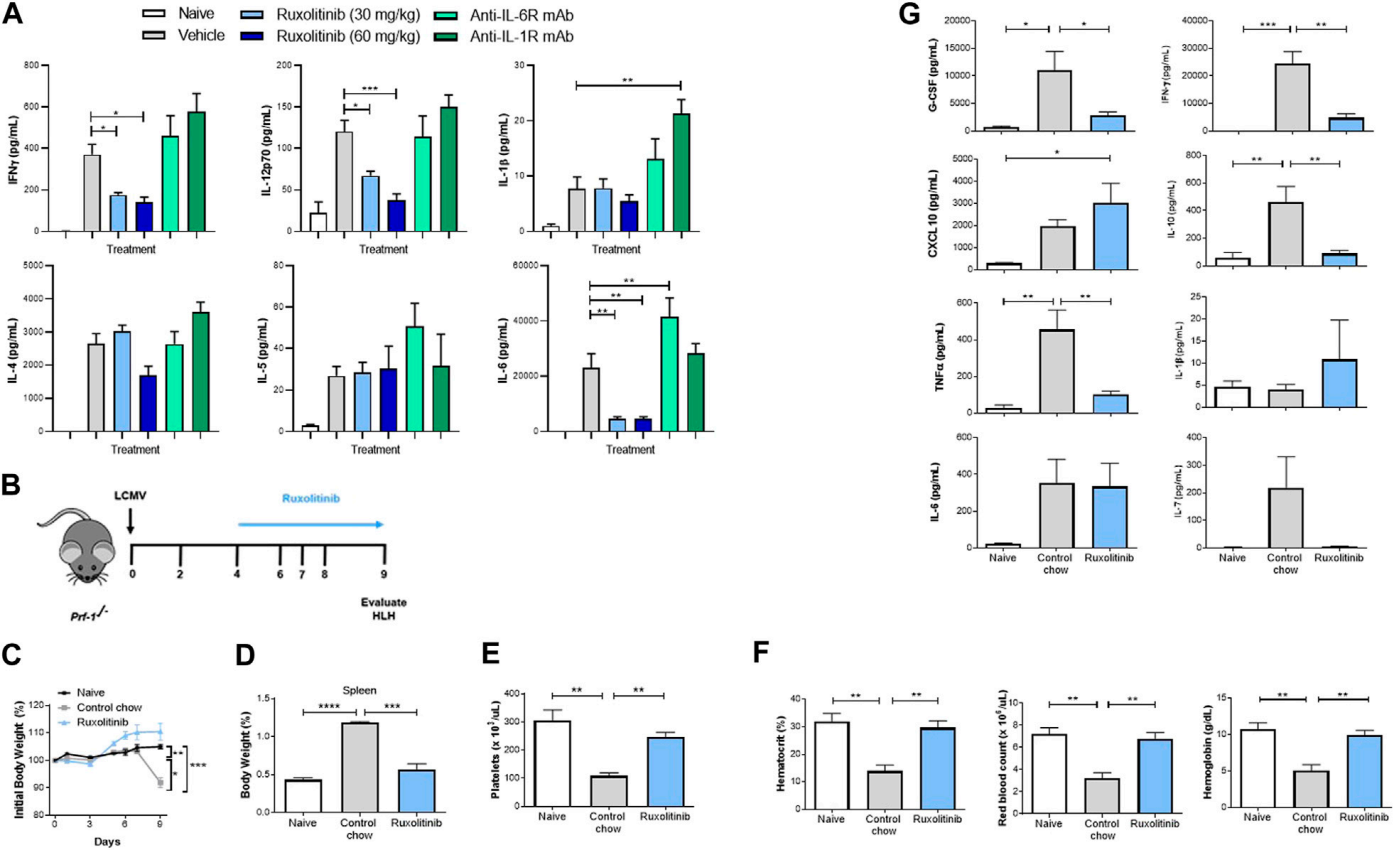
Ruxolitinib lowers cytokine levels in murine models of acute inflammation **(A)** BALB/c mice were challenged with Con-A and dosed 30 min later with vehicle control, 30 or 60 mg/kg of ruxolitinib, anti-IL-1 mAb (25 mg/kg), or anti-IL-6R mAb (25 mg/kg). Animals were sacrificed 120 min later, and serum was collected. Multiplex cytokine analysis was performed to quantify proinflammatory cytokine concentrations **(B)** *Prf1*−/− mice were infected with LCMV and treated with either control chow or ruxolitinib chow (ruxolitinib) starting on day 4 post-infection. Mice were euthanized on day 9 post-infection **(C)** Kinetics of body weight percentage in treatment-naive, control chow-treated, and ruxolitinib-treated mice **(D)** Spleen percentage of body weight in treatment-naive, control chow-treated, and ruxolitinib-treated mice **(E,F)** Blood analysis showing number of PLTs, HCT, levels of HB, and RBC counts **(G)** Levels of serum cytokines. *N* = 5 animals per group. Data are representative of three independent experiments. *p < 0.05, **p < 0.01, ***p < 0.001, ****p < 0.0001.

Cytokine production is the hallmark of CSS, and the majority of the implicated cytokines signal through the JAK/STAT pathway (Albeituni et al., 2019). We therefore examined whether JAK1/2 inhibition with ruxolitinib at doses that mimic clinically achievable human JAK/STAT target inhibition could be used as a therapeutic modality to dampen hyperinflammation without inducing broad immune suppression. First, using the potent T-cell mitogen ConA, we studied an *in vivo* model characterized by broad inflammatory cytokine release and lymphocyte proliferation (Gantner et al., 1995). Similar to individuals experiencing hyperinflammatory syndromes, mice receiving ConA have elevated serum levels of multiple inflammatory cytokines, as well as physiological changes, such as fever, malaise, hypotension, hypoxia, capillary leak, multiorgan toxicity, and potentially death. To study the effect of ruxolitinib in this model, animals were therapeutically dosed with 30 or 60 mg/kg ruxolitinib 30 min post ConA challenge. Two hours after ConA injection animals were sacrificed and serum was collected for cytokine analysis. When compared with vehicle-treated animals, ruxolitinib significantly reduced systemic levels of many CSS-implicated cytokines that signal through the JAK/STAT pathway (e.g., IL-6, IL-12, and IFN-γ) in a dose-dependent manner (Figure 1A). Importantly, other JAK-dependent cytokines did not show a significant reduction (e.g., IL-4 and IL-5), indicating that ruxolitinib treatment downregulated the exaggerated cytokine milieu, but did not induce broad immunosuppression. Ruxolitinib did not have a significant effect on cytokines independent of the JAK signaling pathway (e.g., IL-1β, Figure 1A).

Beyond inhibiting the JAK/STAT pathway, the use of monoclonal antibodies (mAb) such as those targeting IL-1 or IL-6 receptors has been explored as a treatment strategy against CSS (Mehta et al., 2020a). To understand these mechanisms further, some animals were dosed therapeutically with blocking mAb against IL-1R or IL-6R 30 min after ConA challenge. Interestingly, neither anti-IL-1R nor anti-IL-6R treatment was able to significantly reduce cytokine levels in our model (Figure 1A), indicating that inhibiting the JAK/STAT pathway effectively targets multiple inflammatory cytokines and clearly differentiates from strategies that block individual mediators (Meletiadis et al., 2020). While higher plasma levels of IL-6 and/or IL-1 can be attributed to the antibodies blocking the receptor, it is unclear why anti-IL-1R and anti-IL-6R failed to reduce other cytokines levels (Nishimoto et al., 2008).

### Ruxolitinib Reduces Splenomegaly and Hyperinflammation in a Model of Primary Hemophagocytic Lymphohistiocytosis

HLH, the prototypical CSS (Meyer et al., 2020), is a rare and often fatal immune disorder characterized by overwhelming activation of T-cell and macrophage compartments and an autocrine loop of proinflammatory cytokines, resulting in unremitting fever, tissue damage and multiorgan failure. To explore the efficacy of oral ruxolitinib at doses which mimic achievable human JAK/STAT target inhibition in primary (i.e., genetic) HLH, we utilized the well-established LCMV infection model of *Prf1* ^*−/−*^ mice (Albeituni et al., 2019; Das et al., 2016). To define the therapeutic efficacy of JAK inhibition, Prf1^−/−^ mice were treated with phosphate-buffered saline or infected with 2 × 10^5^ PFU LCMV Armstrong (Figure 1B). Beginning on day 4 post-infection, animals were fed with either control or ruxolitinib chow (2 g/kg) to achieve a JAK target inhibition comparable to 60 mg/kg PO, BID ruxolitinib (Incyte Corporation, Wilmington, DE, United States). On day 9 post LCMV infection, coinciding with the peak of T-cell antiviral immune response, animals were euthanized and organs harvested. Consistent with the published literature (Das et al., 2016), LCMV-infected animals developed anemia, splenomegaly and thrombocytopenia (Figures 1C–F and data not shown); however, following treatment with ruxolitinib, these parameters completely normalized (Figures 1C–F). Importantly, ruxolitinib reduced circulating levels of cytokines implicated in the hyperimmune response, such as G-CSF, IFN-γ, IL-10 and TNF-α (Figure 1G). Furthermore, we also detected a significant reduction in both the percentage and total numbers of IFNγ^+^TNFα^−^ and IFNγ^+^TNFα^+^ CD44^+^CD62L^−^CD8^+^ effector memory T-cells in mice treated with ruxolitinib chow (Supplementary Material Figure S1).

### Ruxolitinib Abrogates Splenomegaly and Hyperinflammation in a Mouse Model of Secondary Hemophagocytic Lymphohistiocytosis

**FIGURE 2:**
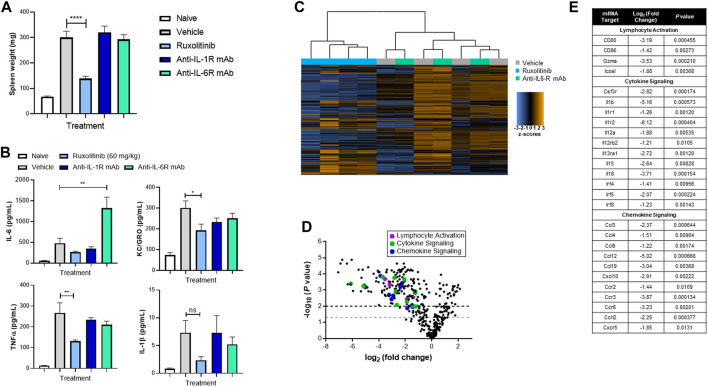
Ruxolitinib abrogates hyperinflammation in a murine model of secondary HLH. C57BL/6 animals were injected with 50 μg of CpG DNA on days 0, 2, 4, 6, and 8. Starting on day 5, corresponding mice were treated with vehicle, ruxolitinib, anti-IL-1R mAb, or anti-IL-6R mAb. On day 9, animals were sacrificed, and spleen size **(A)** and cytokines **(B)** were measured. Data are representative of five independent experiments. *p < 0.05, ***p < 0.001, ****p < 0.0001 **(C)** Heatmap of splenic T-cell mRNA expression profiling panel, with blue indicating downregulation and orange indicating upregulation. Genes below threshold count (<20) were removed **(D)** Volcano plot of splenic T-cell differential gene expression between ruxolitinib and vehicle (baseline) treatment. Points above the gray and black dashed lines indicate adjusted *p* < 0.05 and < 0.01, respectively **(E)** Table highlighting individual differentially expressed proinflammatory genes.

We previously showed that ruxolitinib treatment reduced symptoms in a mouse model of secondary HLH (Das et al., 2016; Albeituni et al., 2019), where hyperinflammation is triggered by immune events as opposed to genetic conditions. Next, we queried whether orally administered doses of ruxolitinib that mimic clinically achievable human JAK/STAT target inhibition would effectively reduce hyperinflammation. As shown in Figure 2A, when compared with vehicle-treated animals, mice receiving 60 mg/kg ruxolitinib had significantly reduced splenomegaly, which coincided with lower levels of pro-inflammatory cytokines KC/GRO (IL-8 functional analogue in rodents) and TNF-α in plasma (Figure 2B) and spleen (data not shown).

Considering that both IL-1 and IL-6 are critical cytokines in CSS, tocilizumab and anakinra have been explored as potential treatments for patients with severe HLH (Dufranc et al., 2020; Mehta et al., 2020a). In our experimental model, treatment with either anti-IL-1R or anti-IL-6R mAb failed to significantly reduce splenomegaly or cytokine levels and indeed dramatically increased plasma levels of IL-6 (Figures 2A,B). These data suggest that blockade of the JAK/STAT pathway may differentiate in efficacy compared with targeting individual cytokines such as IL-1 or IL-6.

### Ruxolitinib Downregulates the T-Cell Inflammatory Transcriptome in a Secondary Hemophagocytic Lymphohistiocytosis Model

To characterize the immunological pathway changes that underpin ruxolitinib-mediated efficacy in the murine secondary HLH model, transcriptome profiling using RNA isolated from splenic T-cells was performed. Principle component analysis revealed clustering of the expression profiles of T-cells from vehicle and anti-IL-6R−treated mice, while ruxolitinib-treated animals clustered separately (Figure 2C). This analysis confirmed that ruxolitinib, but not anti-IL-6R, treatment induced discrete changes in gene expression in HLH mice. Differential expression analysis further revealed that ruxolitinib downregulated many proinflammatory genes, including those involved in lymphocyte activation, cytokine signaling and chemokine signaling (Figure 2D). This analysis resulted in a list of 27 genes that we then analyzed for putative upstream regulators of gene expression (Figure 2E). Consistent with the known physiological mechanism of action of ruxolitinib (Spoerl et al., 2014; Verstovsek et al., 2017; Gozzetti et al., 2020; Zeiser et al., 2020), we confirmed that genes implicated in the response to IL-1 and IFN, as well as several T-cell trafficking chemokines, were the top gene regulators inhibited in ruxolitinib-treated mice.

### Ruxolitinib Lowers Cytokine Production and Proliferation Without Impairing Degranulation

**FIGURE 3:**
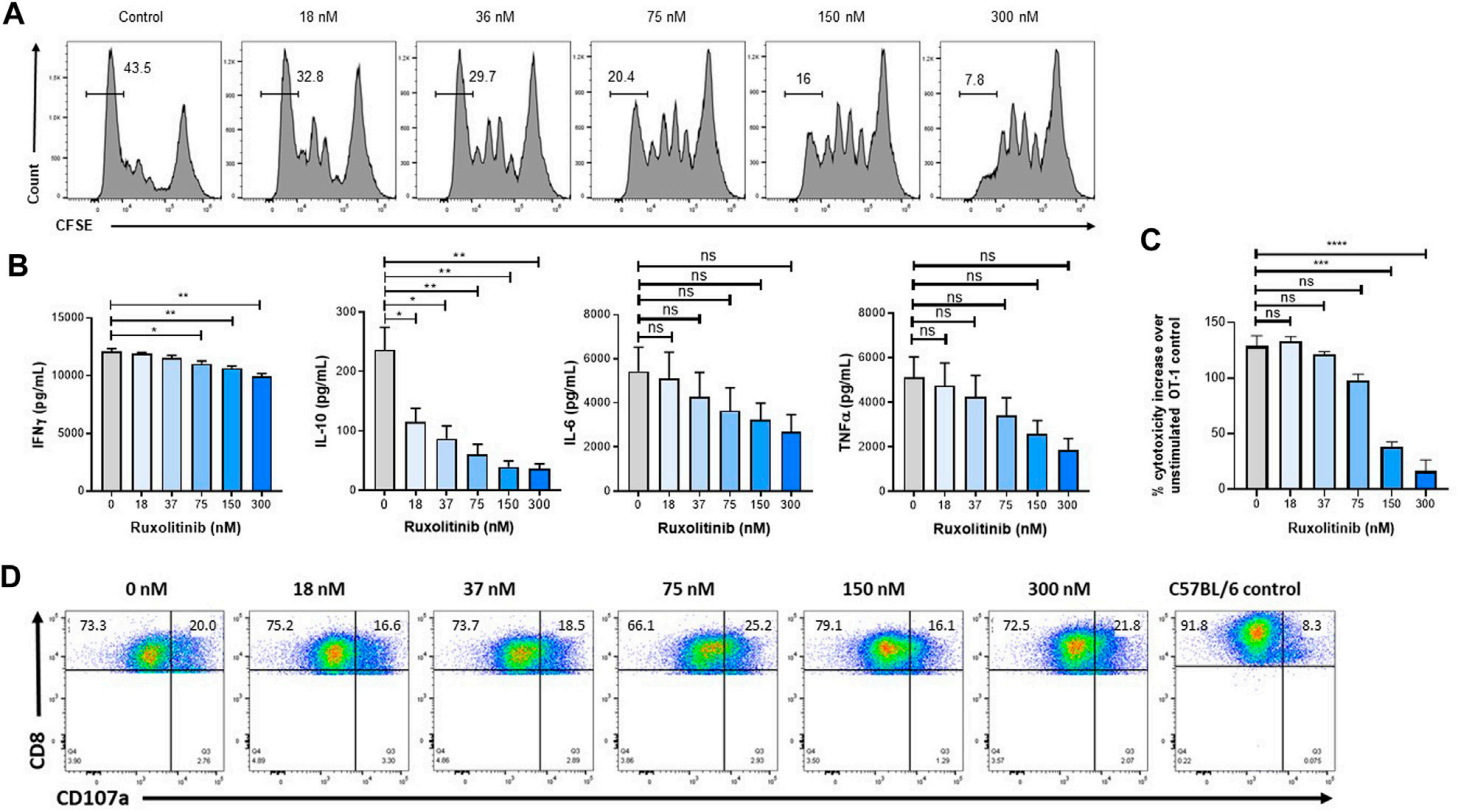
Ruxolitinib reduces proliferation without affecting effector function of T-cells. To measure OT-1 CD8^+^ T-cell expansion, CFSE-labeled splenocytes were stimulated with SIINFEKL peptide and increasing ruxolitinib concentrations **(A)** Four days later, the number of divisions was calculated by flow cytometry **(B)** Supernatants were collected from the cultures and cytokines were quantified **(C)** To measure degranulation, OT-1 cultures were stimulated overnight with OVA-expressing EG-7 cells, and CD107a levels were measured by flow cytometry **(D)** OT-1 cultures were activated with OVA peptide and incubated with OVA-expressing EG-7 cells transfected with a luciferase plasmid, with luciferase activity being measured to quantify cytotoxicity. Data are representative of two independent experiments. *p < 0.05, **p < 0.01. **p < 0.001. ns indicates not significant.

Because activated T-cells play an important antitumor role, the effect of ruxolitinib on T-cell expansion and effector function was studied. As a model for antigen-specific proliferation and cytolytic activity, we used the well-established OT-1 system, where lymphocytes express a transgenic TCR VαVβ5 specific for the OVA257–264 peptide (SIINFEKL) and restricted to H-2Kb. Antigen-specific T-cells were obtained from OT-1 spleens and expanded *in vitro* with anti-CD3/CD28 antibodies. As seen in Figure 3A, only concentrations of ruxolitinib higher than the *in vitro* IC_50_ (in the single-digit nanomolar range (Quintás-Cardama et al., 2010)) result in a reduction in proliferation, as measured with CFSE staining by flow cytometry (Quah et al., 2007). Importantly, ruxolitinib induced a significant and concentration-dependent reduction on the levels of several inflammatory cytokines implicated in CSS hyperactivity (e.g., IFN-γ and IL-10, with non-significant trends in IL-6 and TNF-α) (Figure 3B).

We then measured the ability of OT-1 antigen-specific lymphocytes to kill target cells after being activated in the presence of ruxolitinib. OT-1 splenocytes activated in the presence of increasing doses of ruxolitinib only saw a reduction in lytic activity at the highest concentrations of ruxolitinib (Figure 3C). Next, we measured the capacity of OT-1 antigen-specific lymphocytes to degranulate in response to OVA-expressing EG-7 tumor cells. As seen in Figure 3D, even T-cells expanded at the higher ruxolitinib concentrations (300 nM) were able to upregulate CD107a in response to the OVA antigen (Betts et al., 2003), indicating that ruxolitinib does not have an adverse effect on the capacity of antigen-specific T-cells to degranulate upon tumor cell encountering. As a negative control, C57BL/6 splenocytes did not degranulate when cocultured with OVA-expressing EG-7 tumor cells (Figure 3D). Therefore, while ruxolitinib treatment reduced proliferation rates and cytokine production of lymphocytes, it did not affect the lytic activity.

### Ruxolitinib Significantly Decreases IL-6 Production by Macrophages

**FIGURE 4:**
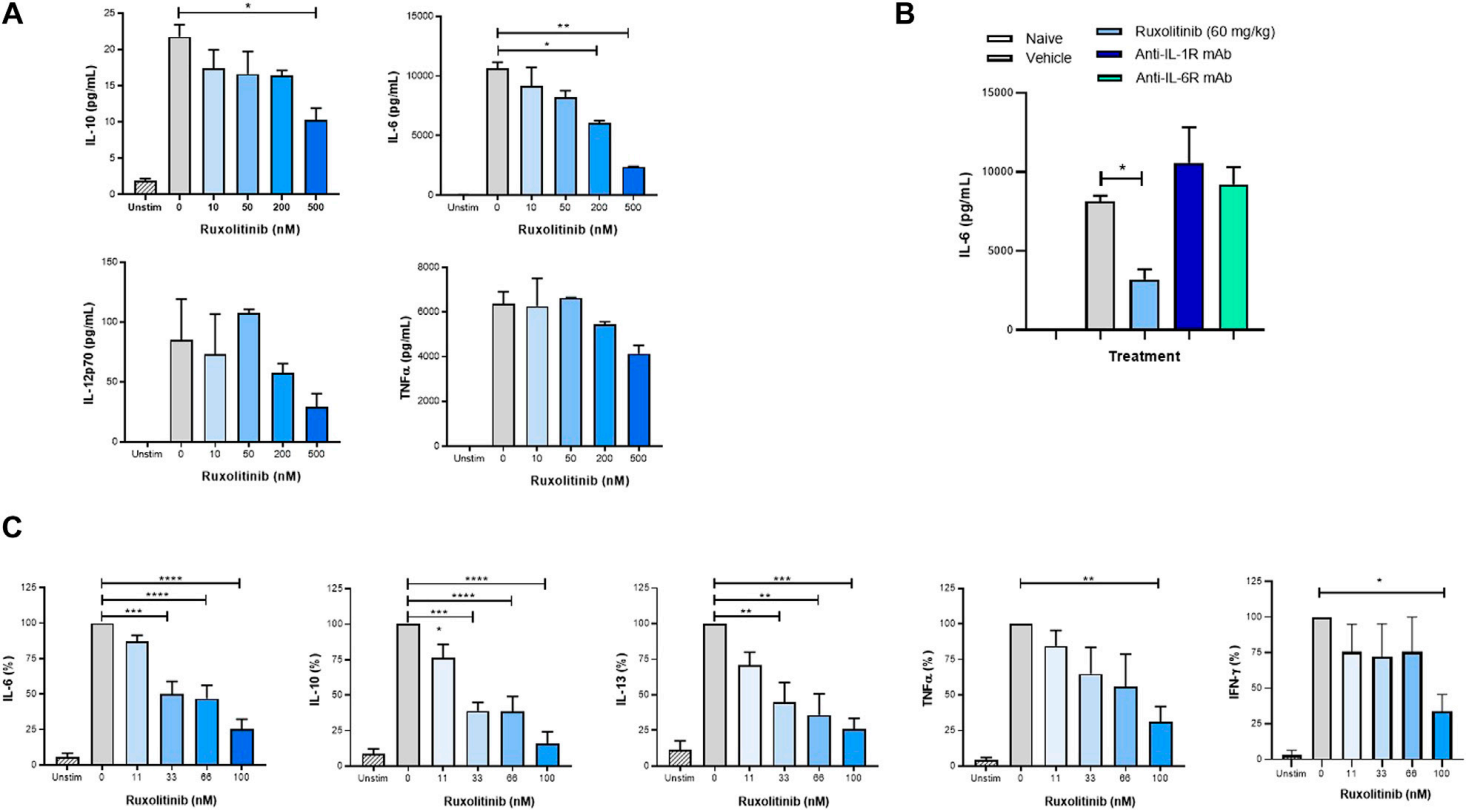
Ruxolitinib lowers cytokine production by murine macrophages and human PBMCs **(A)** Bone marrow–derived macrophages were expanded *in vitro* with granulocyte-colony stimulating factor, and ruxolitinib was added to the cultures 24 h in advance of being activated with 5 ng/ml LPS. Culture supernatants were collected 24 h post-LPS stimulation, and multiplex cytokine analysis was performed **(B)** C57BL/6 mice were prophylactically dosed with vehicle, ruxolitinib, anti-IL-1R, or anti-IL-6R for 3 days before intraperitoneal LPS injection. Cytokines were measured from peritoneal lavages 2 h after injection. Data are representative of two independent experiments. *p < 0.05, **p < 0.01 **(C)** PBMCs from healthy volunteers were stimulated *in vitro* with anti-CD3 and anti-CD28 antibodies and increasing concentrations of ruxolitinib. Seven days later, supernatants were collected, and cytokines were measured by multiplex cytokine analysis. Data are normalized to individual donors.*p < 0.05.

Along with T-cells, macrophages are the main cellular subset implicated in hyperinflammatory conditions (Giavridis et al., 2018; Norelli et al., 2018; Crayne et al., 2019). We therefore investigated the effect of JAK1/2 inhibition on cytokine production by macrophages. First, murine bone marrow–derived macrophages were expanded *in vitro* with M-CSF, and ruxolitinib was added to the cultures on day 6. Treatment with ruxolitinib reduced IL-6 production following stimulation with LPS in a concentration-dependent manner, indicating that the pharmacologic activity of ruxolitinib in reducing production of inflammatory cytokines is not exclusive to T-cells (Figure 4A). We also observed a non-significant trend toward reduction in IL-12p70 and TNF-α (Figure 4A).

We then queried whether ruxolitinib, anti-IL-1R, or anti-IL-6R mAbs would effectively reduce *in vivo* cytokine production by peritoneal macrophages. Mice were prophylactically treated with vehicle, ruxolitinib (PO), anti-IL-1R (IP), or anti-IL-6R (IP) for 3 days before receiving an intraperitoneal LPS injection. Two hours after LPS injection, cytokines were measured from peritoneal lavage fluid. Only ruxolitinib-treated animals showed a significant reduction in IL-6 levels (Figure 4B), as well as a trend toward IL-12 reduction (data not shown). The inability of anti-IL-6R to reduce IL-6 levels is consistent with clinical studies that have noted an increase in serum IL-6 levels in patients with Castleman disease (Nishimoto et al., 2008), CAR-T cells (Quintás-Cardama et al., 2010) or COVID-19 (Luo et al., 2020) treated with tocilizumab.

### Ruxolitinib Reduces Cytokine Production by Human PBMCs

To explore the translational validity of the mouse studies, T-cells were obtained from freshly isolated human PBMCs of healthy adults. Following activation with anti-CD3/CD28−coated beads (Richman et al., 2018) and treatment with ruxolitinib, concentrations of several inflammatory cytokines were measured after 7 days of culture. As seen in Figure 4C, when compared with DMSO control, concentrations of ruxolitinib associated with the cellular IC_50_ significantly reduced levels of key CSS cytokines (IL-6, IL-10, IL-13, TNF-α and IFN−γ).

Taken together, our preclinical studies using doses of ruxolitinib that mimic clinically achievable JAK/STAT target inhibition demonstrate profound downregulation of the proinflammatory cytokine milieu implicated in CRS and CSS pathophysiology. These experimental findings may have implications for the treatment of primary and secondary HLH (Jianguo et al., 2020), as well as other hyperinflammatory conditions (Capochiani et al., 2020; Del Valle et al., 2020; Karki et al., 2020; Yeleswaram et al., 2020).

## Data Availability

The raw data supporting the conclusions of this article will be made available by the authors, without undue reservation.

## Abbreviations

mAb, monoclonal antibodies; PFU, plaque-forming unit; LCMV, lymphocytic choriomeningitis virus; CFSE, carboxyfluorescin succinimidyl ester; WT, wild type; M-CSF, macrophage colony-stimulating factor; IFN, interferon; G-CSF, granulocyte-colony stimulating factor; Con-A, Concanavalin-A; IP, intraperitoneal; IV, intravenous; BID, twice daily, QD, once daily; DMSO, dimethyl sulfoxide.
