# Supplementary figures and images for: Ruxolitinib, a JAK1/2 Inhibitor, Ameliorates Cytokine Storm in Experimental Models of Hyperinflammation Syndrome
Source: Front Pharmacol. 2021 Apr 22;12:650295. doi: 10.3389/fphar.2021.650295 (PMC8107823; doi:10.3389/fphar.2021.650295)

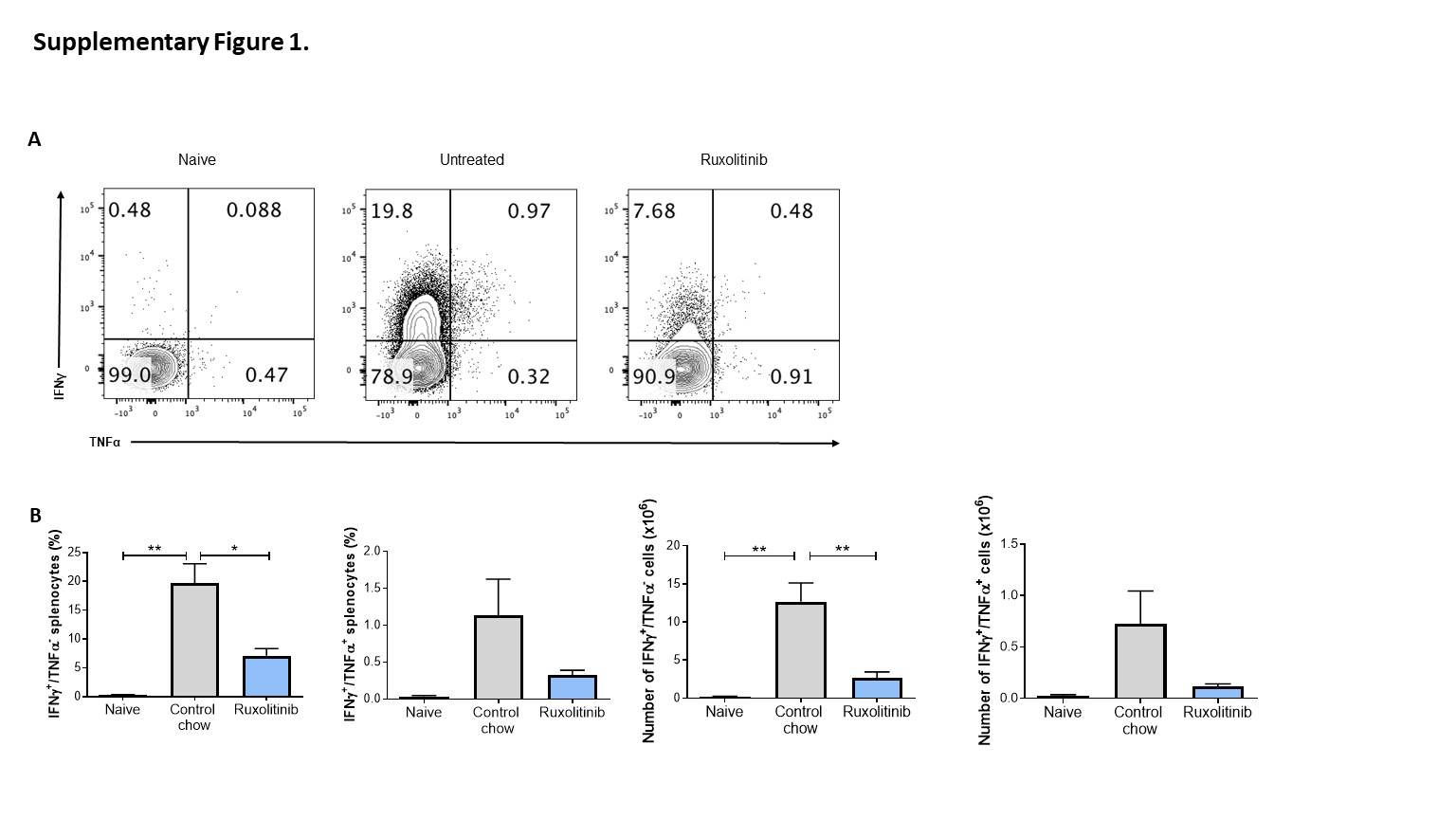

Supplement: Supplementary file 1 [file image1.jpeg]
